# Systems analysis of MVA-C induced immune response reveals its significance as a vaccine candidate against HIV/AIDS of clade C

**DOI:** 10.1186/1742-4690-9-S2-P303

**Published:** 2012-09-13

**Authors:** C Gómez, B Perdiguero, V Jimenez, A Filali-Mouhim, K Ghneim, E Haddad, E Quakkerlaar, J Delaloye, A Harari, T Roger, T Duhem, R Sekaly, C Melief, T Calandra, F Sallusto, A Lanzavecchia, R Wagner, G Pantaleo, M Esteban

**Affiliations:** 1 Centro Nacional de Biotecnología, Madrid, Spain; 2 VGTI, Port Saint Lucie, FL, USA

## Background

Based on the partial efficacy of the HIV/AIDS Thai trial (RV144) with a canarypox vector prime and protein boost, attenuated poxvirus recombinants expressing HIV-1 antigens are increasingly sought as vaccine candidates against HIV/AIDS.

## Methods

Here we describe using systems analysis the biological and immunological characteristics of the attenuated vaccinia virus Ankara strain expressing the HIV-1 antigens Env/Gag-Pol-Nef of HIV-1 of clade C (referred as MVA-C).

## Results

MVA-C infection of human monocyte derived dendritic cells (moDCs) induced the expression of HIV-1 antigens at high levels from 2 to 8 hpi and triggered moDCs maturation as revealed by enhanced expression of HLA-DR, CD86, CD40, HLA-A2 and CD80 molecules. Infection ex vivo of purified mDC and pDC with MVA-C induced the expression of immunoregulatory pathways associated with antiviral responses, antigen presentation, T cell and B cell responses. Similarly, human whole blood or primary macrophages infected with MVA-C express high levels of proinflammatory cytokines and chemokines involved with T cell activation. The vector MVA-C has the ability to cross-present antigens to HIV-specific CD8 T cells in vitro and to increase CD8 T cell proliferation in a dose-dependent manner. The immunogenic profiling in mice after DNA-C prime/MVA-C boost combination revealed activation of HIV-1-specific CD4 and CD8 T cell memory responses, that are polyfunctional and with effector memory phenotype. Env-specific IgG binding antibodies were also produced in animals receiving DNA-C prime/MVA-C boost.

## Conclusion

Our systems analysis of profiling immune response to MVA-C infection highlights the potential benefit of MVA-C as vaccine candidate against HIV/AIDS for clade C, the prevalent subtype virus in the most affected areas of the world.

